# Biological activities (antimicrobial, anti-inflammatory, anti-coronavirus (229E)) and chemical composition of some essential oils

**DOI:** 10.1038/s41598-026-48032-1

**Published:** 2026-04-24

**Authors:** Gehan E. Gab-Allah, Ahmed Elsayed Abouelwafa, Sahar W. M. Hassan

**Affiliations:** 1 Biological and Geological Sciences Department, Faculty of Education, Alexandria University, Alexandria, Egypt; 2 National Institute of Oceanography and Fisheries (NIOF), Cairo, Egypt

**Keywords:** Essential oils, Antimicrobial, Anti-inflammatory, Antiviral, GC–MS, Biochemistry, Biotechnology, Drug discovery, Microbiology

## Abstract

Microbial drug resistance represents one of the major causes of death today. Traditional medicine provides an important health care service and can be applied as alternative therapy. The growing interest in essential oils (EOs) has led to extensive research, due to their diverse applications in pharmaceuticals, food products and cosmetics. The current study evaluated the antimicrobial activity of menthol, lemon, clove and camphor EOs against *Staphylococcus aureus, Staphylococcus epidermidis, Escherichia coli, Enterococcus faecalis, Bacillus subtilis, Pseudomonas aeruginosa*, *Candida albicans* and *Aspergillus niger*. The tested EOs exhibited a broad spectrum of antimicrobial activity with inhibition zone diameter ranged from 11 to 22 mm. The highest inhibition was observed against *P. aeruginosa* and *A. niger*. The anti-inflammatory activity of the EOs was also assessed and was as 64.5 ± 4.1%, 10 ± 1%, 10 ± 2.1% and 7.5 ± 3.1% for menthol, camphor, lemon and clove EOs, respectively. Examination of antiviral activity of the EOs against Coronavirus 229E showed varied antiviral potency with IC_50_ values of 16.248, 9.90, 4.645 and 2.256 μg/mL for camphor, menthol, clove and lemon, respectively. Chemical constituents of menthol and camphor essential oils were detected using gas chromatography coupled with mass spectrometry (GC–MS). Results indicated the dominance of eucalyptol (29.17%), camphor (7.96%) in camphor EO and 9,12-Octadecadienoic acid (Z,Z), (12.24%), levomenthol (10.94%) in menthol EO samples.

## Introduction

Humans have fought against infectious diseases since ancient times. A noteworthy number of deaths are caused by antimicrobial resistant strains, and predictions indicate that this number may reach 20 million by 2050^1,2^. The discovery of antibiotic utilization for treating bacterial diseases was in 1923. However, over time, the overuse and misuse of antibiotics have contributed to the development and spread of antibiotic-resistant microbes^3^. Viral infections are major contributors for several human diseases, extending from self-limiting conditions to those accountable for over 20% of worldwide deaths^1,4^.

Common spoilage fungal species including *Penicillium* and *A. niger* significantly reduce the quality and shelf life of different food products^5–7^. Fungicides and synthetic chemical preservers are usually used to inhibit their growth. However, their potential toxicity and development of resistant strains represent major public health concern^8^. Subsequently, there is an increasing demand for safer and natural preservation alternatives^6,8,9^. An alternative to synthetic preservers is the use of natural antimicrobial compounds extracted from plants, including plant extracts and essential oils.

Essential oils are aromatic, oil-based compounds mainly extracted from plants through distillation^10–12^. These compounds play a key role in the defense strategies against microbial infections. The usage of essential oils can be traced back to Ancient Egypt, where they were obtained by infusing plant materials in animal fats and vegetable oils. Currently, essential oils are used to manage different health illnesses such as cancer, stress, pain and other infections. Essential oils are therapeutically applied via oral consumption, topical application and aromatic inhalation. Most existing studies have focused on their antimicrobial effects when used directly in liquid form. Though, there is only restricted scientific research on the antimicrobial properties of their airborne volatile compounds.

Natural essential oils show considerable potential as active agents against bacteria, fungi, and viruses which cause different other health threats. As a result of their easy accessibility, widespread availability, and low toxicity, they have gained global attention^13^. Essential oils from thyme, cinnamon, oregano and clove have proven to have substantial antimicrobial effects against a range of pathogens. These natural components are progressively preferred as food preservative due to their consumer preference, effectiveness, and safety^14^.

The essential oils are reported as anti-inflammatory agents acting to suppress LPS-induced inflammatory responses in macrophages through interfering with JNK signaling, NF-κB and ERK^15^. Moreover, essential oils can be distributed as aerosols, making them valuable as air purifiers and disinfectants which could play a key role in combating pandemics like COVID-19^13^. While viral diseases have long been a subject of scientific interest^16^, the latest global COVID-19 epidemic has significantly strengthened investigation focus in this area^17–19^.

The aim of the current study offers perspectives on the use of menthol, clove, camphor and lemon essential oils (EOs) in fighting microbial pathogens and contributes to the existing understanding of their possible effectiveness. Moreover, anti-inflammatory and antiviral activities of EOs were also evaluated as a trial to offer ecofriendly, economic and safe compounds for different applications.

## Materials and methods

### Essential oils

Four essential oils were utilized in this study: camphor (*Cinnamomum camphora*) oil, clove (*Syzygium aromaticum*) oil, lemon (*Citrus limon*) oil and menthol (*Mentha arvensis*) oil. The essential oils were purchased from two certified suppliers in Egypt: Imtenan Natural Products Company (Egypt) and El-Captin CO. All oils were of food- pharmaceutical-grade quality, supplied with certificates of analysis, and stored at 4 °C in airtight amber glass containers until use to prevent degradation of volatile constituents. The botanical names and oil designations of the used essential oils are displayed in Table 1.

**Table 1:** The botanical names and oil designations.

Oil designation	Botanical names	Essential oil
Camphor	*Cinnamomum camphora*	Camphor
Eugenol	*Syzygium aromaticum*	Clove
Limonene	*Citrus limon*	Lemon
Menthol	*Mentha arvensis*	Menthol

### Antimicrobial activity

#### Preparation of indicator pathogenic microorganisms

To evaluate the antimicrobial potential of essential oils, several pathogenic microorganisms were utilized. These included Gram-positive (*S. epidermidis* ATCC 12228, *S. aureus* ATCC 6538, *E. faecalis* ATCC 29212 and *B. subtilis* ATCC 6633) and Gram-negative bacteria (*E. coli* ATCC 8739 and *P. aeruginosa* ATCC 9027) as well as yeast strain (*C. albicans* ATCC 10231) and fungal strain (*A. niger* ATCC 16404). They were obtained from National Institute of Oceanography and Fisheries, Alexandria. Bacterial inoculums were prepared by suspending cultures directly in Mueller–Hinton broth (Himedia, India), adjusting the turbidity to an absorbance of 0.5 at 600 nm, Yeast suspensions were prepared in PDA broth (Merck, Germany), with absorbance adjusted to 0.5 at 600 nm.

#### Agar well diffusion technique

To estimate the antimicrobial activity of the essential oils, agar-well diffusion technique was used. The microbial suspension (50 µl) was inoculated in Mueller–Hinton agar plates (Himedia, Thane, India). After the surface drying, sterile stainless-steel tools were used to punch wells with 6 mm diameter into the agar. 50 μL of the corresponding sample was added to each well. The plates were incubated at 37 °C for 24 h. All samples were tested in triplicate and afterward, the diameters of the clear inhibition zones around the wells were measured in millimeters^20^.

### Anti-inflammatory activity

To assess the capability of essential oils for preventing protein denaturation, a modification of the previously described method was employed^21^. In this procedure, 0.1 mL of albumin was mixed with 1.9 mL of phosphate-buffered saline (PBS, pH 6.4). Then 1 mL of each essential oil was separately added. For the negative control, an equal volume of distilled water replaced the essential oil. The resultant solutions were incubated at 37 °C for 20 min. After incubation, samples were heated at 70 °C for 5 min. Once cooled, the absorbance of each mixture was measured at 660 nm using a spectrophotometer (Evolution 60S, USA). Matching concentration of a standard anti-inflammatory agent (Diclofenac sodium) was used and exposed to similar treatment for comparative study. All samples were tested in triplicate.

The percentage inhibition of protein denaturation was calculated using the formula:

$$\% {\text{ Inhibition}} = \left[ {\left( {{\text{Abs sample}}{-}{\text{Abs control}}} \right)/{\text{Abs control}}} \right] \times {1}00$$

where “Abs” refers to absorbance values. The concentration required to achieve 50% inhibition (IC_50_) was gained by plotting a dose–response curve.

### Cytotoxicity assays

Vero E6 cells (Cytion catalog number 305008, Germany) were seeded into culture plates at a density of 2 × 10^4^ cells per well one day before viral infection. After exposing the cells to the virus, the medium containing serial dilutions was removed, and the cells were washed with a phosphate-buffered saline solution. The crystal violet assay was employed to evaluate viral infectivity by measuring the inhibition of cytopathic effects (CPE) and estimating cell viability^22^. 0.1 mL volume of diluted essential oil solution, containing virus stock at a concentration of 1.0 × 10⁶ CCID₅₀, was added to the mammalian cells. This concentration was chosen to produce noticeable CPEs within 48 h of infection. The cytotoxic (CC₅₀) and antiviral inhibitory (IC₅₀) concentrations were estimated using the GraphPad PRISM software (San Diego, USA).

#### Antiviral activity

Antiviral activity was carried out at (Nawah-Scientific, Egypt) and evaluated against Coronavirus 229E (Gained from Nawah-Scientific, Egypt). To treat the cells, 100 µl of culture medium encompassing the desired concentration of the test oil was applied. Antiviral effectiveness was evaluated through a sequence of two-fold dilutions, beginning with an initial concentration of 1000 µg/ml. Two control sets were involved: virus-infected cells without treatment (viral control) and uninfected cells (cell control). The culture plates were incubated for 72 h at 37 °C in an atmosphere containing 5% CO₂. The progress of cytopathic action (CPE) was detected under a light microscope throughout the incubation period. Antiviral activity against Coronavirus 229E was assessed relying on the method defined by Pauwels et al.^23^. From these remarks, the concentration necessary to inhibit 50% of the cytopathic action (IC_50_) was assessed.

### GC–MS characterization of the essential oil

An Agilent Technologies gas chromatograph (model 7890B) paired with a mass spectrometer detector (model 5977A) was set up on the GC–MS system in the main lab of Nawah Scientific Inc. (Mokatam, Cairo, Egypt). The sample was prepared by dissolving it in dichloromethane. A DB-624 capillary column (30 m long, 320 µm internal diameter, 1.8 µm film thickness) was applied in the GC study. The temperature program began at 40 °C (held for 1 min), then raised to 70 °C per minute until reaching 250 °C, which was held for 5 min. Hydrogen was used as the carrier gas, flowing at 3.0 ml/min, with a split ratio of 1:20 and an injection volume of 1 µl. The temperatures of both the injector and detector were maintained at 250 °C. Mass spectrometry was directed using electron ionization (EI) at 70 eV, scanning a mass-to-charge (m/z) range of 30 to 440, with a solvent delay of 360 s. Compounds were recognized by comparing the obtained fragmentation patterns to those in the Wiley and NIST mass spectral libraries^24^.

## Results and discussion

### Antimicrobial action of different essential oils (EOs)

**Fig. 1:**
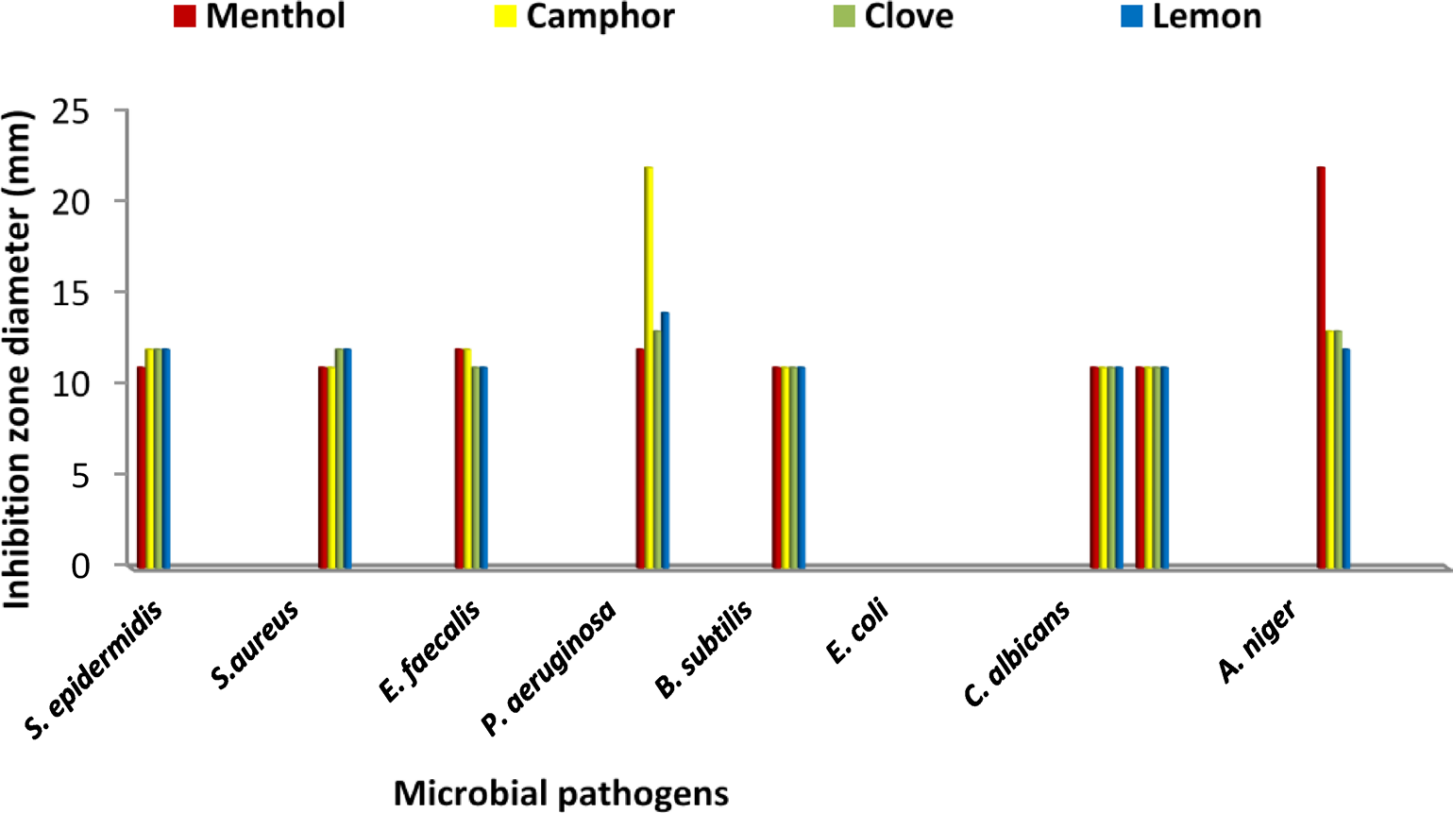
Antimicrobial activity (expressed as inhibition zone diameter (mm)) of the tested essential oils against pathogenic microbes.

The antimicrobial activity of EOs was evaluated against the tested pathogenic microbial species. Results of EOs antimicrobial activity (Fig. 1) indicated broad spectrum of antimicrobial activity for all EOs against the tested pathogens. Also, the results of the antimicrobial assay indicated that menthol and camphor EOs exhibited the highest antimicrobial activity. The antimicrobial activity for both EOs ranged from 11 to 22 mm. Menthol EO recorded the highest activity against *A. niger* (22 mm), while camphor oil exhibited the highest activity (22 mm) against *P. aeruginosa*. Generally, more antimicrobial action of EOs was observed toward Gram positive bacteria than Gram negative bacteria. The same observation was detected by Rathore et al.^25^ and Duda-Madej et al.^26^ who reported more effectiveness of EOs against Gram-positive bacteria compared to Gram-negative species. Opposite results were reported by Naik et al.^13^. Essential oils (EOs) exhibit antimicrobial activity primarily through the disruption of microbial membrane integrity, which increases cell permeability and can lead to cell lysis. Unlike conventional antibiotics, EOs can act on different cellular targets.

Antibacterial potential of menthol was confirmed by Zhao et al.^27^ against MARSA with inhibition zone diameter (IZ) of 11.9 mm. In vitro works revealed that menthol suppressed the bacterial growth with inhibition zones range (10.0–25.3 mm) against *Streptococcus pyogenes*, *S. mutans*, *S. faecalis*, *Lactobacillus acidophilus* and *S. aureus* and the yeast *C. albicans* with zone of inhibition as 7.1–18.5 mm^28^. The action mechanism is guessed to include disruption of the membrane^29^. Antimicrobial activity of menthol against *E. coli* was reported recording growth rate reduction by approximately 50%. These results confirmed the significant action of menthol on bacterial adaptation and replication through disruption of membrane-associated characteristics by reduced H^+^-flux through the membranes^30^.

The recorded broad antibacterial activity of camphor is constituent with Abdollahi et al.^31^ who examined the antibacterial effect of nanogel containing camphor showing a complete suppression of *E. coli*, *Listeria monocytogenes*, *S. aureus* and *P. aeruginosa* at different concentrations. Other studies have highlighted the role of camphor as a component of essential oils from different sources and recorded its antibacterial effectiveness toward broad range of Gram negative and Gram-positive bacteria in addition to inhibition of *C. albicans*^31^. Comparable investigation by Laczkowski et al.^32^ confirmed the bioactivity of camphor derivatives against different bacteria and fungi.

In accordance with our results, camphor displayed potent bioactivity against* E. faecalis*, *S. aureus* and *S. mutants*^33,34^. EO from camphor showed a significant reduction influence on *E. coli* biofilm after 30 min of contact as was previously reported^35^. Other studies confirmed the antimicrobial activity of camphor EO against *Choanephora cucurbitarum*, *S. aureus* and *E. coli*^36,37^. It uses different actions such as disruption of double-layer structure of the cell membrane and interference with membrane-bound proteins and enzymes^35,38–41^.

Clove and lemon EOs displayed less antimicrobial activity with IZ diameter ranged from 11 to 13 mm for clove and 11 to 14 mm for lemon, respectively. A study by Abers, et al.^42^ reported low antimicrobial activity of lemon, while absence of antimicrobial activity was detected for clove against *S. epidermidis*. Nevertheless, some studies confirmed the antimicrobial activity of clove essential oil, along with its primary active compound eugenol^43,44^. Clove also exhibited antimicrobial activity against S.* aureus* and *E*.* coli*^45^.

A study^46^ confirmed the antimicrobial activity of lemon against *S. aureus*. Out of the 15 bacterial isolates tested, the lemon EO effectively suppressed the growth of 13 bacterial strains with inhibition zones between 10 and 30 mm. *Pseudomonas aeruginosa* and *S. epidermidis* were the only strains that showed complete resistance to the extract^47^. The volatilization, medium’s solubility, diffusion rate and agar may influence the size of inhibition zones.

### Anti-inflammatory activity

Protein denaturation is a known contributor to inflammation. Protein denaturation can trigger an inflammatory response by generating auto-antigens, which are key contributors to the onset of chronic inflammation. The anti-inflammatory potential of the tested essential oils was determined using the heat-induced albumin denaturation assay. All samples were tested in triplicate, and the results are listed in Table 2. Menthol oil exhibited the highest inhibitory effect, with an average inhibition of 64.5 ± 4.1 followed by camphor and lemon exhibiting inhibition of 10 ± 1 and 10 ± 2.1. In contrast, clove oils exhibited less inhibitory activity (7.5 ± 3.1%). The restricted effectiveness in this assay may result from a low concentration of active compounds, lack of synergistic interactions, or instability of constituents during processing. Variations in phytochemical structure, extraction methods, and compound stability may affect the anti-inflammatory action. The ability of these natural compounds to stabilize proteins is likely due to the presence of polyphenols and their metabolites, which may influence inflammatory signaling pathways^48,49^. The anti-inflammatory outcome also may be due to the presence of terpenoids, flavonoids, tannins, steroids and alkaloids^50,51^ or by inhibiting pro-inflammatory cytokines production and regulating Th2-mediated inflammation^52^. The anti-inflammatory effect of menthol was reported in previous studies^53–58^.

**Table 2:** Effect of essential oils on protein denaturation.

Essential oil	% Inhibition (Mean ± SD)
Menthol	64.5 ± 4.1
Lemon	10±2.1
Camphor	10 ± 1.0
Clove	7.5 ± 3.1
Diclofenac standard	97.32±1.19
Control	0

Yang et al.^15^ investigated the anti-inflammatory potential of essential oils from peels of 21 citrus species through measuring the expression levels of key inflammatory markers and cytokines. Notably, lemonen essential oils exhibited the strongest anti-inflammatory activity by significantly suppressing inflammatory mediators and cytokine production in RAW264.7 cells treated with lipopolysaccharide. Limonene has been shown to possess anti-inflammatory effects across multiple disease models, potentially by downregulating pro-inflammatory cytokines^59,60^ and modulating key signaling pathways such as p38 MAPK, NF-κB, JNK, and ERK^61,62^. Different researches have displayed that EO from lemon can modulate anti-inflammatory efficacy^63–66^.

### Antiviral activity

**Fig. 2:**
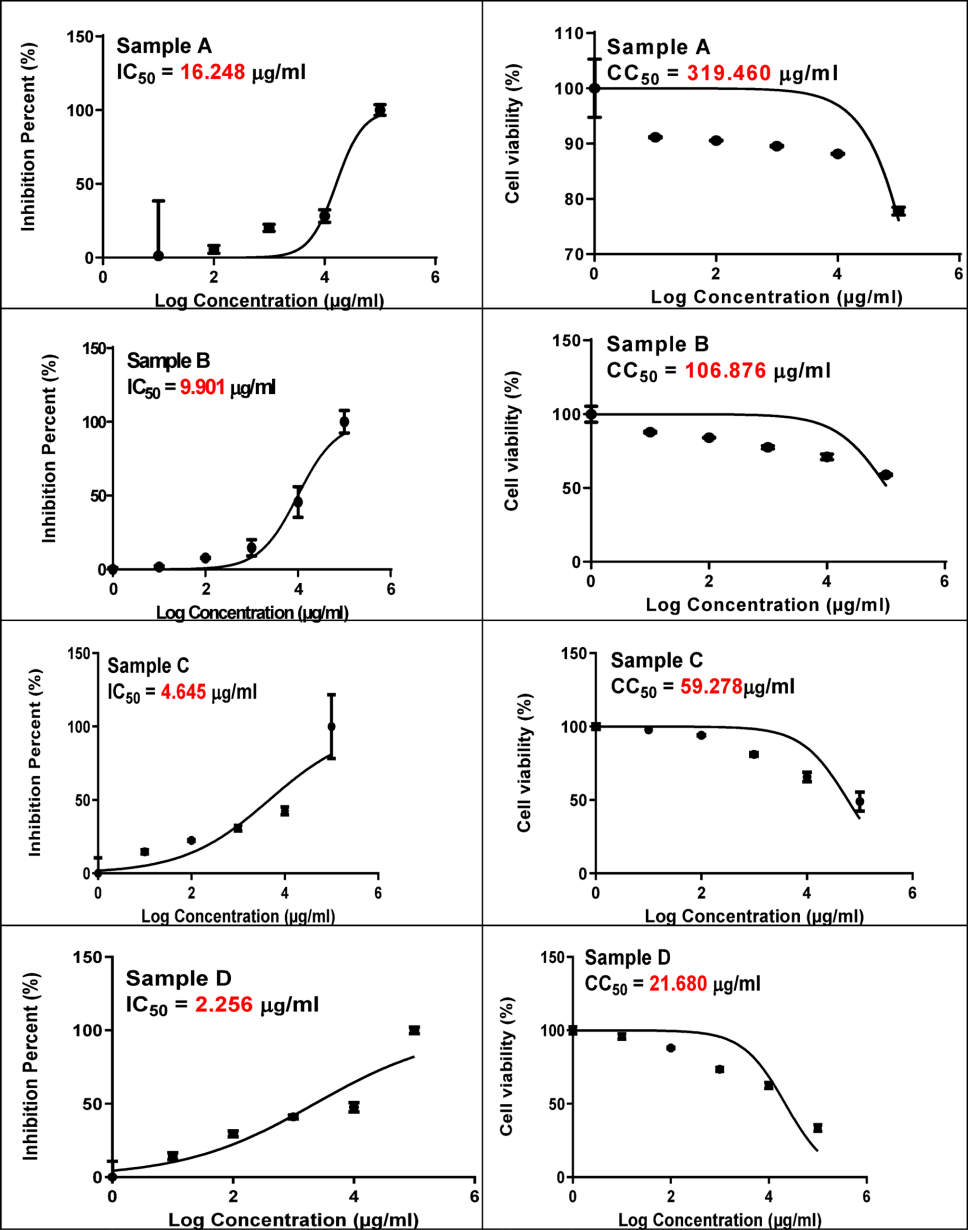
Effect of (**A**) Camphor, (**B**) Menthol, (**C**) Clove and (**D**) Lemon essential oils on viability percentage of Vero E6.

To determine whether the essential oil directly reduced viral infectivity and progeny production via cytotoxic effects, the viability of Vero E6 cells was evaluated after incubation in media with or without the oil. EOs were examined as antiviral agents against Coronavirus 229E. The estimated IC_50_ is the concentration of EO inhibiting 50% of the target virus, while CC_50_ is the concentration of EO killing 50% of host cells and the selectivity index (SI) represents CC_50_/IC_50_ and reflects the drugs’ safety. Accordingly, Lower values of IC_50_ indicate higher antiviral activity, while higher SI values indicate safety of the used compounds. Results (Fig. 2) showed that camphor EO exhibited a relatively high CC₅₀ value of 319.460 μg/mL, with an IC_50_ of 16.248 μg/mL, yielding a selectivity index (SI) of 19.6. Menthol EO demonstrated a CC₅₀ of 106.876 μg/mL and an IC_50_ of 9.901 μg/mL, indicating potent antiviral potency with moderate cytotoxicity and corresponding to SI of 10.8. Clove EO showed a CC_50_ of 59.278 μg/mL and a notably low IC_50_ of 4.645 μg/mL, with an SI of 12.7. Lemon EO exhibited the highest antiviral potency (IC_50_ value of 2.256 μg/mL), but its cytotoxicity was relatively higher (CC_50_ = 21.680 μg/mL), resulting in a lower selectivity (SI of 9.6). Thus, lemon > clove > menthol > camphor according to antiviral potency (IC_50_), while selectivity (SI) indicated that camphor > clove > menthol > lemon. Camphor and menthol were chosen for further study. Menthol and camphor were reported as antiviral agents^67,68^.

Essential oils of lemon encompass active compounds like citral exhibiting antiviral potential against H1N1, HSV-2, and Adenovirus (ADV) type 40^69^. Clove was proven as rich source of active compounds such as eugenol exhibiting immunostimulatory and antiviral activities^70^. Lemon and clove were also reported as antiviral sources targeting SARS-CoV-2^71^. The antiviral effect of essential oils is primarily due to their capability to block viral attachment to host cells and interfere with membrane fusion, thereby disrupting the assembly and stability of macromolecules on the viral surface, they also can hinder structural rearrangements, and ultimately blocking viral entry^69,72,73^.

### Gas chromatography–mass spectrometry (GC–MS) analysis

**Fig. 3:**
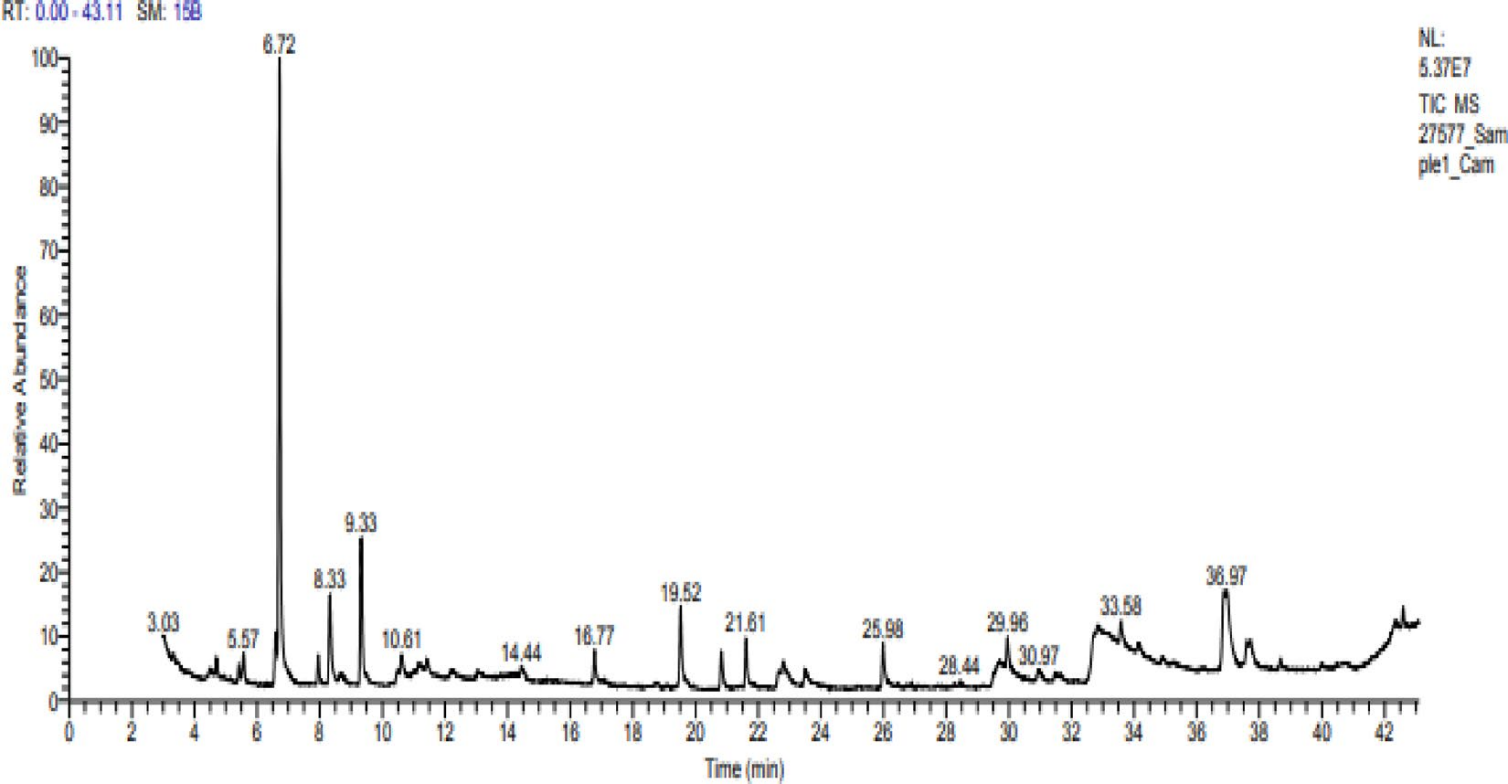
GC-MS chromatogram of camphor essential oil.

**Fig. 4:**
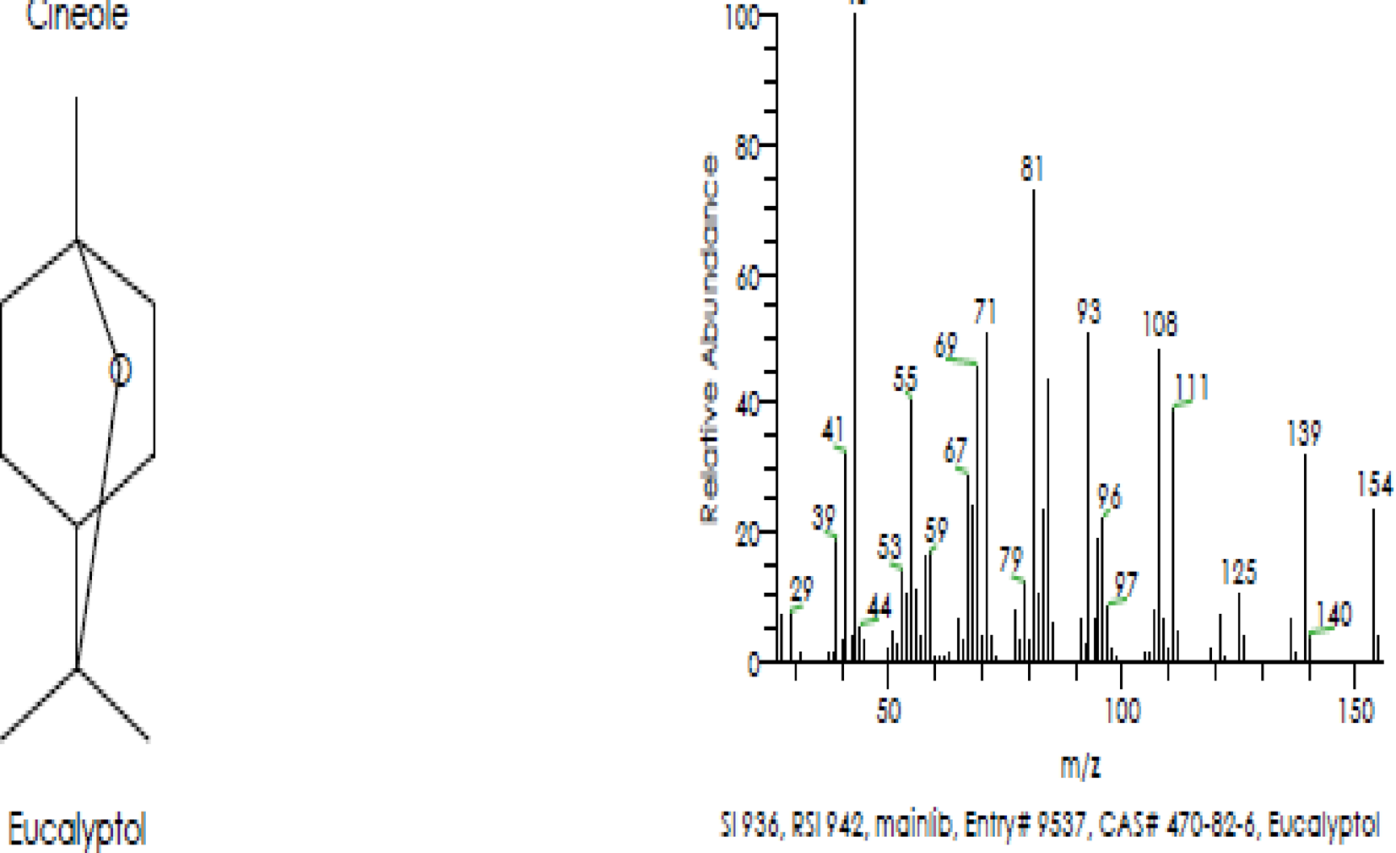
Spectrum of the major compound (eucalyptol) in camphor essential oil.

**Fig. 5:**
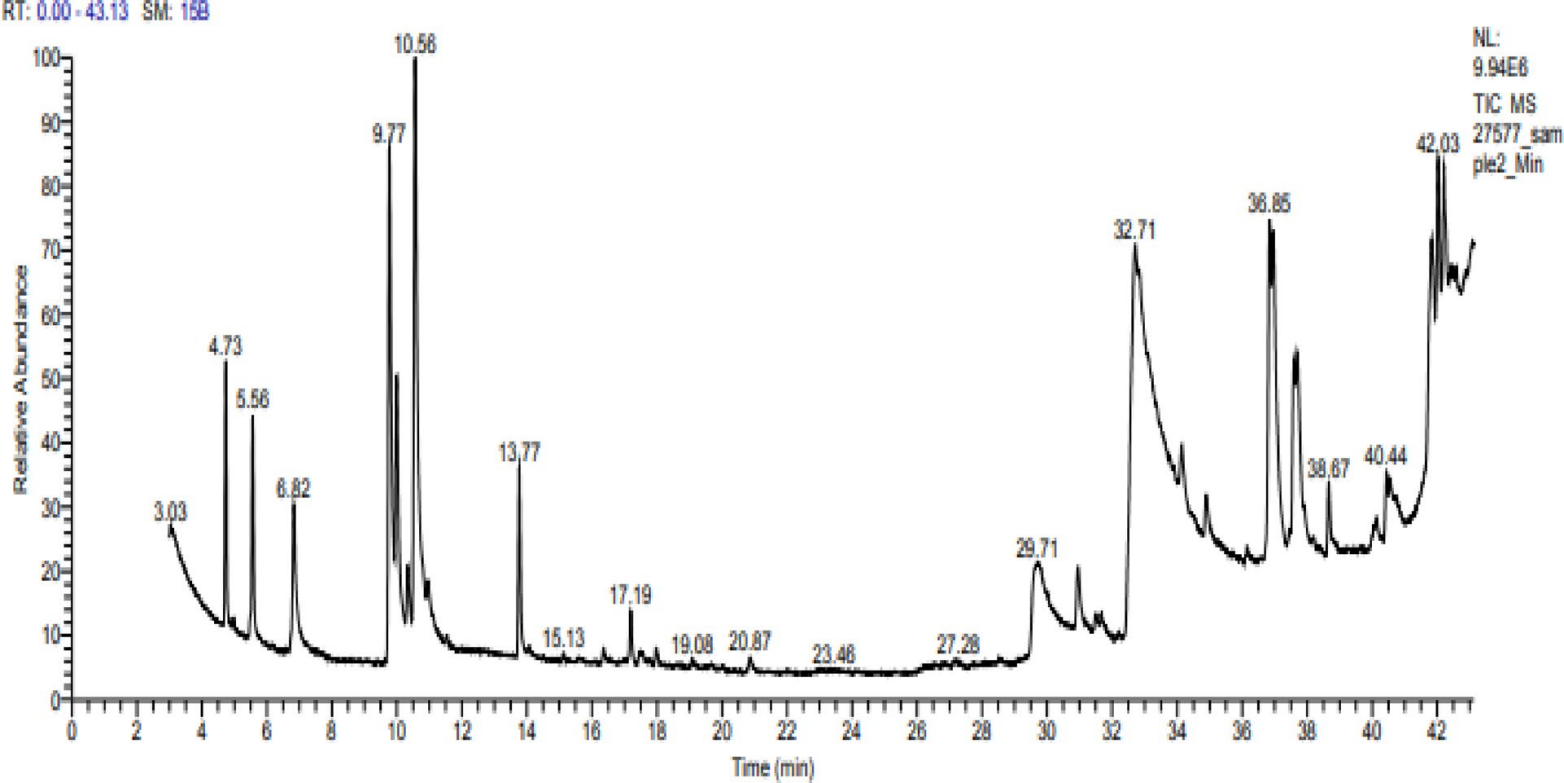
GC-MS chromatogram of menthol essential oil.

**Fig. 6:**
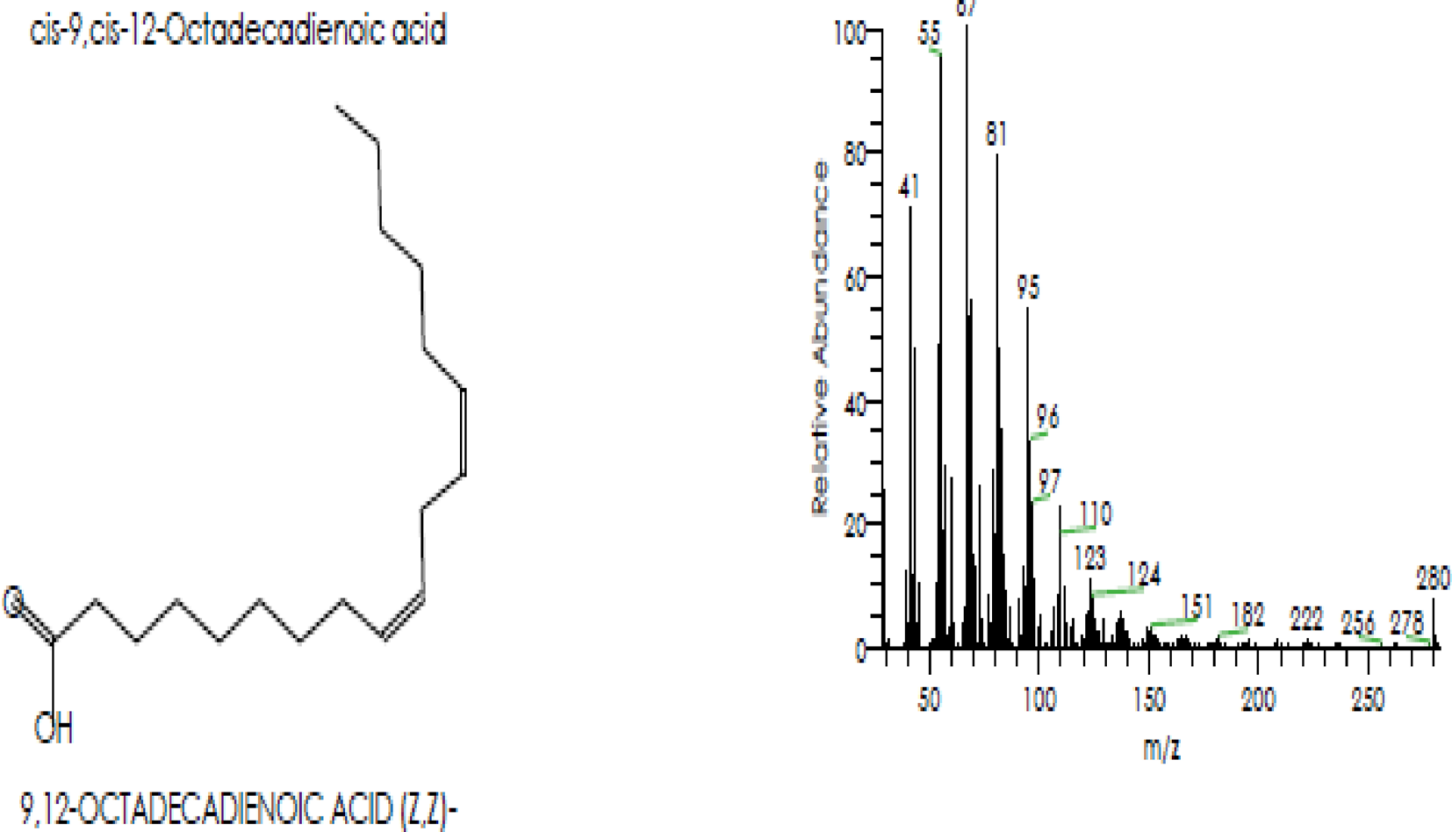
Spectrum of the major compound (12-Octadecadienoic acid (Z,Z)) in menthol essential oil.

Camphor and menthol essential oils were selected for further characterization study using GC–MS to detect the components of both essential oils. The GC–MS analysis of Camphor oil displayed roughly major 6 compounds as shown in (Table 3 & Fig. 3) eucalyptol (29.17%), Camphor (7.96%), Acetaldehyde, (3,3-dimethylcyclohexylidene), (E)-Acetaldehyde (4.88%), Oleic acid (2.71%), o-Cymene (1.92%) and Fenchone (1.64%). Figure 4 displays spectrum of the major compound (eucalyptol). On the other hand, there were 4 principal components in Menthol essential oil as indicated in (Table 4 & Fig. 5) representing 9,12-Octadecadienoic acid (Z,Z) (12.24%); Levomenthol (10.94%), 1-Menthone (9.61%); 9,12-Octadecadienoic acid (Z,Z), 2-hydroxy-1-(hydroxymethyl) ethyl ester (6.94%). Figure 6 represents spectrum of the major compound (9,12-Octadecadienoic acid (Z,Z).

**Table 3:** GC-MS of chemical constituents of Camphor essential oil.

Retention time (min)	Chemical compound	Molecular weight (g/mol)	Molecular formula	Area (%)	Structural formula
6.71	Eucalyptol	154	C₁₀H₁₈O	29.17	
9.32	Camphor	152	C₁₀H₁₆O	7.96	
8.32	Acetaldehyde, (3,3-dimethylcyclohexylidene)-, (E)-Acetaldehyde	152	C₁₀H₁₆O	4.88	
29.96	Oleic Acid	282	C₁₈H₃₄O₂	2.71	
6.59	o-Cymene	134	C₁₀H₁₄	1.92	
7.95	Fenchone	152	C₁₀H₁₆O	1.64	

**Table 4:** GC-MS of chemical constituents of Menthol essential oil.

Retention time (min)	Chemical compound	Molecular weight (g/mol)	Molecular formula	Area (%)	Structural formula
32.70	9,12-Octadecadienoic acid (Z,Z)	280	C₁₈H₃₂O₂	12.24	
10.55	Levomenthol	156	C₁₀H₂₀O	10.94	
9.76	1-Menthone	154	C₁₀H₁₈O	9.61	
36.84	9,12-Octadecadienoic acid (Z,Z), 2-hydroxy-1-(hydroxymethyl)ethyl ester	354	C₂₁H₃₈O₄	6.94	

GC–MS of camphor EO showed that eucalyptol was the major compound with peak area (29.17%). The bioactivity of eucalyptol was proven in previous studies. Moukhfi et al.^74^ showed that eucalyptol displayed antibacterial action against strains of* Salmonella* and *E. coli* with MIC range from 0.35 to 11.37 mg/ml. A recent study reported the antibacterial potential of natural thymol–eucalyptol-based mixture toward *Pseudomonas *sp., *Salmonella *sp., *Clostridium perfringens* and *E. coli*^75^. This thought is reliable with recognized research revealing that eucalyptol trigger seepage of cellular substances, in addition to disrupting vital metabolic processes^76–78^.

The antifungal efficiency of eucalyptol was evaluated indicating that eucalyptol had marked effect against some clinical dermatophyte isolates^79^. Other investigations confirmed the antifungal effect of eucalyptol^80–82^ through its action on fungal morphology and cell membrane^83^. Eucalyptol was reported for its anti-inflammatory activity treating signs of respiratory infections, pancreatitis, colitis and common cold^84,85^. Also, eucalyptol decreased the inflammation in ankle tissues by inhibiting inflammasome activation of NLRP3^86^.

In accordance with the present results, eucalyptol was reported as antiviral agent^87,88^. Antiviral activity of eucalyptol contained in Camphor was also confirmed in previous studies^89–91^. Antiviral action of heterocyclic alkaloid‑like compounds from camphoric acid against influenza virus was previously proved^68^. Another investigation showed that camphor- based1,3-thiazolidin-4-one and thiazole derivatives and the thiazolidin-4-one derivatives, compound 8b have antiviral efficiency against vaccinia virus with IC_50_ values range 2.4–3.7 μM and 9.5 μM, respectively with moderate cytotoxicity for both compounds.

On the other hand, GC–MS chromatogram of menthol EO showed that 9,12-Octadecadienoic acid (Z,Z) was the major compound with peak area of 12.24%. The bioactivity of 9,12-Octadecadienoic acid was indicated in other previous studies. Kapoor et al.^92^ screened the antimicrobial activity of 9, 12-octadecadienoic acid derivatives showing good results against *E. coli, S. epidermidis, S. aureus, P. aeruginosa*, *B. subtilis*, *A. niger* and *C. albicans*. In accordance with our results, the same finding was recently reported by Onoabedje et al.^93^ who reported the broad spectrum of 9,12-octadecadienoic acid antimicrobial potential toward the test pathogens including* S. typhimurium*,* E. coli*, *S. pyogenes, S. aureus, C. albicans* and *A. niger* with inhibition zones diameter of 0–20 mm.

Anti-inflammatory activity of 9, 12-Octadecadienoic acid (Z,Z) methyl ester was reported in previous investigations^94–97^. Anti-inflammatory activity of the major compound in levomenthol was previously reported by Wade et al.^98^. In a study, the octadecanoic acid, methyl ester has displayed its antiviral action against measles disease with very low cytotoxicity against Vero cell line recording more than 90% cell viability^99^. The antiviral activity of octadecanoic acid contained in the menthol essential oil was reported in different investigations^67,99,100^.

### Infrared spectroscopy (IR) of camphor and menthol essential oils

**Fig. 7:**
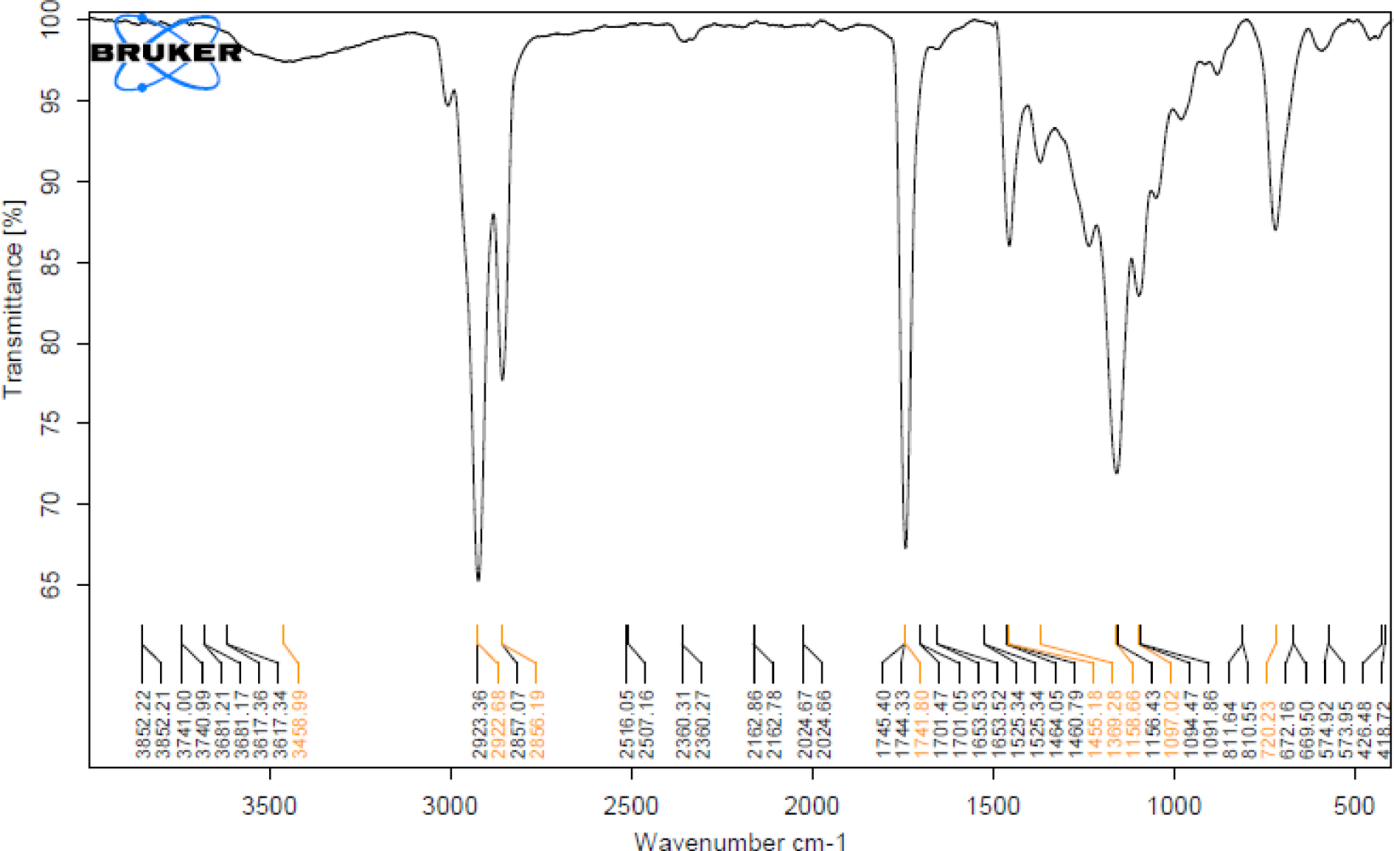
IR Spectra of camphor essential oil.

Camphor and menthol essential oils w﻿ere further characterized by Fourier-transform infrared (FTIR) spectroscopy to detect the functional groups. Regarding IR of camphor (Fig. 7), it showed a broad peak at 3458.98 cm^−1^ assigned to O–H stretching (in enol form), sharp bands at (2923.36–2856.19 cm^−1^) representing C–H stretching, a sharp C=O stretching band at (1744.33 cm^−1^) and C-H stretching band (in enol form) at (1741.80 – 1455.18 cm^−1^) region. Nearly the same functional groups were observed in a study by Desheesh et al.^101^. On the other hand, menthol exhibited C–H stretching bands from 2922.55 to  2856.74 cm^−1^. These function groups are approximate to those indicated in a study by Surapaneni et al.^102^.

**Fig. 8:**
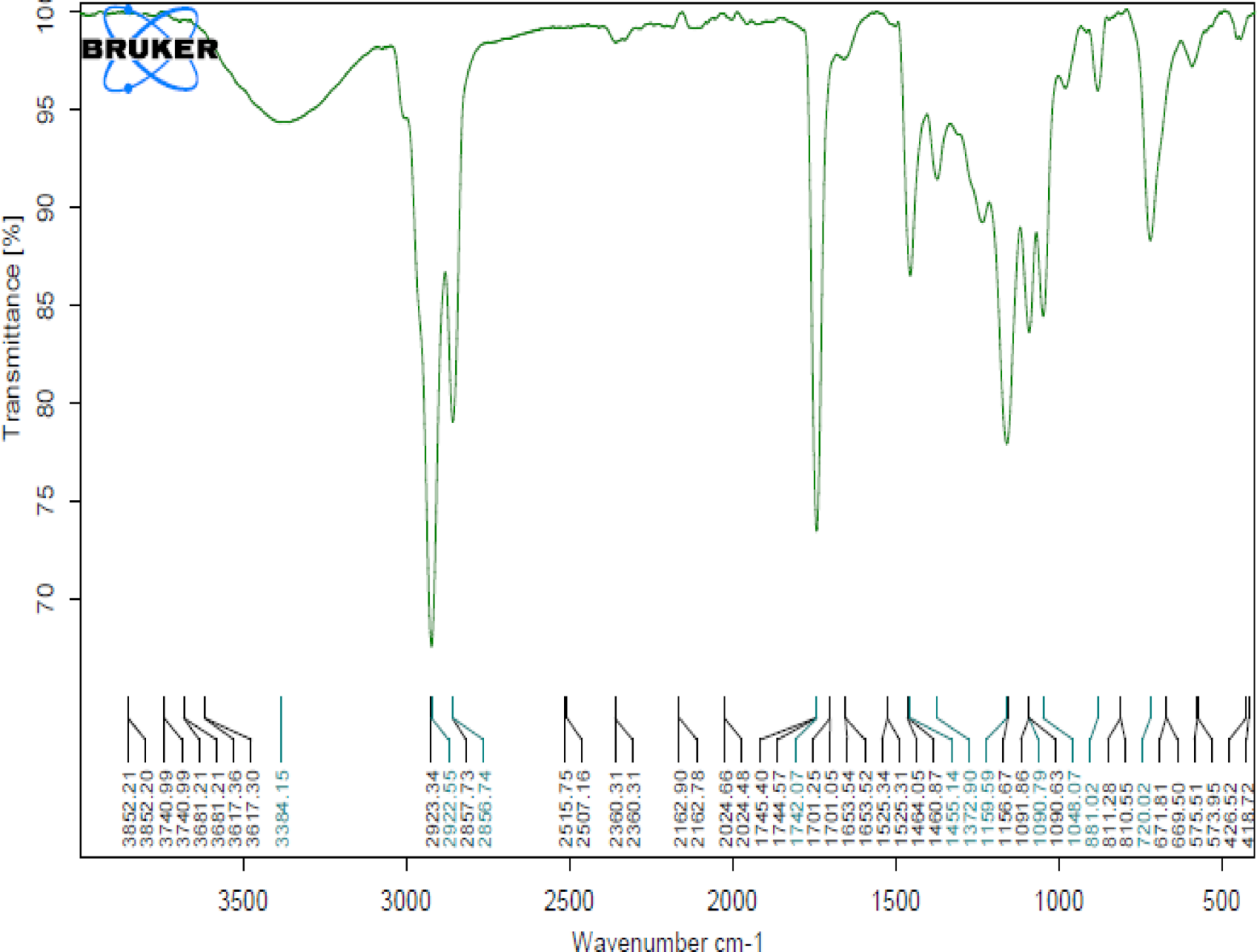
IR Spectra of menthol essential oil.

Future studies concerning detection of the antimicrobial activity of EOs by estimating the minimum inhibitory concentration (MIC) of each used essential oil, and evaluation of anti-inflammatory and antiviral activities of the tested EOs will be conducted in vivo models. Moreover characterization and detection of the active components in lemon and clove essential oils also will be directed (Fig. 8).

## Conclusion

The results of this investigation revealed the promising prospective of using menthol, camphor, lemon and clove essential oils as antimicrobial agents with sufficient suppressive effects against Gram positive, Gram negative bacteria, pathogenic fungi and yeast species. Essential oils showed also moderate anti-inflammatory and anti-Coronavirus 229E effects. Overall results support the application of essential oils from natural sources as an ecofriendly, safe and economic biological agents.

## Data Availability

The datasets generated and analyzed during the current study are available from the corresponding author on reasonable request.
